# Oil and gas production and spontaneous preterm birth in the San Joaquin Valley, CA: A case–control study

**DOI:** 10.1097/EE9.0000000000000099

**Published:** 2020-06-05

**Authors:** David J. X. Gonzalez, Allison R. Sherris, Wei Yang, David K. Stevenson, Amy M. Padula, Michael Baiocchi, Marshall Burke, Mark R. Cullen, Gary M. Shaw

**Affiliations:** a Emmett Interdisciplinary Program in Environment and Resources, Stanford University, Stanford, California; b Department of Pediatrics, School of Medicine, Stanford University, Stanford, California; c Department of Obstetrics, Gynecology & Reproductive Sciences, University of California at San Francisco, San Francisco, California; d Department of Epidemiology and Population Health, School of Medicine, Stanford University, Stanford, California; e Department of Earth System Science, School of Earth, Energy and Environmental Sciences, Stanford University, Stanford, California; f Department of Medicine, School of Medicine, Stanford University, Stanford, California.

**Keywords:** Preterm birth, Birth outcomes, Oil and gas, Ambient air pollution

## Abstract

Recent studies report an association between preterm birth and exposure to unconventional oil and gas wells. There has been limited previous study on exposure to conventional wells, which are common in California. Our objective was to determine whether exposure to well sites was associated with increased odds of spontaneous preterm birth (delivery at <37 weeks).

**Methods:**

We conducted a case–control study using data on 27,913 preterm birth cases and 197,461 term birth controls. All births were without maternal comorbidities and were located in the San Joaquin Valley, CA, between 1998 and 2011. We obtained data for 83,559 wells in preproduction or production during the study period. We assessed exposure using inverse distance-squared weighting and, for each birth and trimester, we assigned an exposure tertile. Using logistic regression, we estimated adjusted odds ratios (ORs) for the association between exposure to well sites and preterm birth at 20–27, 28–31, and 32–36 weeks.

**Results:**

We observed increased ORs for preterm birth with high exposure to wells in the first and second trimesters for births delivered at ≤31 weeks (adjusted ORs, 1.08–1.14). In stratified analyses, the associations were confined to births to Hispanic and non-Hispanic Black women and to women with ≤12 years of educational attainment. In a secondary analysis, we found evidence that exposure to wells in preproduction is associated with higher concentrations of particulate matter.

**Conclusions:**

We found evidence that exposure to oil and gas well sites is associated with increased risk of spontaneous preterm birth.

## Abstract 2

Supplemental Digital Content is available in the text.

**WHAT THIS STUDY ADDS**

An estimated 17.6 million US residents live in close proximity to oil and gas wells, including 2.1 million Californians. However, the health effects of living in proximity to new and active well sites are not well characterized. We examined whether exposure to well sites was associated with preterm birth risk. We conducted a case–control study in the San Joaquin Valley, CA, an area with the most intensive oil and gas production activity in California, predominantly with conventional methods. We observed an association between preterm birth and exposure to oil and gas well sites. In a secondary analysis, we found evidence of higher concentrations of ambient air pollutants at air monitoring sites in proximity to drilling sites compared with unexposed monitors.

An estimated 17.6 million people in the United States live within 1.6 km (1 mile) of an active oil or gas well, including 2.1 million California residents.^1^ The United States recently became the leading global producer of petroleum and natural gas and drilling activity has correspondingly increased, including in California, which is among the most productive states for crude oil.^2,3^ Previous studies have found associations between spontaneous preterm birth and exposure to environmental contaminants, as well as other factors, including stress psychosocial stress, genetics, infection, and race.^4,5^ However, the relative contribution of each of these factors has not been well characterized. Preterm birth, defined as delivery before 37 weeks of gestation, increases risk of infant morbidity and mortality.^6,7^ Preterm births are described as spontaneous for women who present with premature labor with cervical dilation or rupture of membranes, and as medically indicated when induced by a care provider due to health complications.^6^

Several recent studies report that women exposed to unconventional hydraulically fractured wells have increased risk of adverse birth outcomes, including preterm birth.^8–10^ There is limited evidence of associations between adverse birth outcomes and conventional oil and gas extraction operations, which comprise the majority of wells in California.^11^ Preproduction and production activities in both conventional and unconventional wells emit contaminants that may impact perinatal health.^1,12–14^ The preproduction stage, which typically is completed in several weeks, includes preparation of the well pad, road construction, drilling, and well completion.^1,12–14^ The production stage may last as long as several decades, though the intensity of production, as well as emissions of ambient air pollutants, may vary throughout the life of an active well.^1^ Recent studies have found that wells in preproduction emit higher concentrations of air pollutants than wells in the production stage per unit time.^15,16^ In addition to air pollutants, residents near well sites may also be exposed to contaminated water, as well as higher noise pollution and community disruption, which have been associated with increased psychosocial stress.^13,17–20^

This study investigated the potential association between spontaneous preterm birth and exposure to oil and gas wells in preproduction or production the San Joaquin Valley, CA. We used a case–control design to assess the association between exposure to well sites and odds of preterm birth.

## Methods

### Study population

We obtained data on live births from eight counties in the San Joaquin Valley, CA: Fresno, Kern, Kings, Madera, Merced, San Joaquin, Stanislaus, and Tulare (Figure). We chose to focus on the San Joaquin valley region because it accounts for the majority of oil and gas production in California. During the study period, oil and gas operations in the San Joaquin Valley comprised 82.7% of wells in preproduction (new wells) and 74.2% of wells in production (active wells), as well as the majority of oil production by volume in the state. The region had a population of approximately 4 million in the 2010 decennial US Census. We obtained data on 892,088 births between 1998 and 2011, comprising all births in nonmilitary hospitals in the study region. From this population, we compiled a dataset that included preterm cases, defined as delivery at fewer than 37 gestational weeks, and term birth controls. Inclusion criteria were singleton births delivered at 20–41 weeks and with a birthweight between 500 and 5,000 g. Of the 771,416 births that fit these criteria, 78,421 were preterm birth cases. We randomly selected 235,263 term birth controls in a 3:1 ratio of controls to cases.

**Figure.**
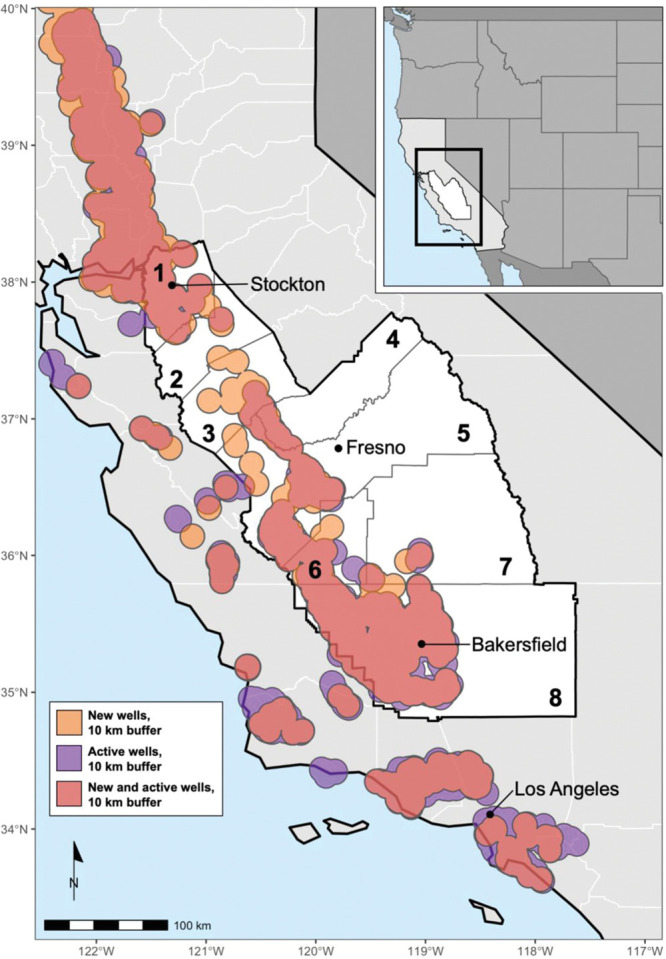
Map of the study region showing 10 km buffers around new wells in preproduction during the study period (orange) and active wells in production during the study period (purple) in California, as well as the overlap between the two (red). The study counties are (1) San Joaquin, (2) Stanislaus, (3) Merced, (4) Madera, (5) Fresno, (6), Kings, (7) Tulare, and (8) Kern.

For all controls and cases, we extracted the residential address at time of birth from the birth certificate. The California Environmental Health Tracking Program Geocoding Service geocoded each address, after standardizing, verifying, and correcting addresses. Geocoding was successful for 221,651 (94.2%) of controls and 73,736 (94.0%) of cases. We linked the cases and controls with discharge data from the Office of Statewide Health and Planning (OSHPD), with successful linkage for 220,137 (99%) controls and 72,907 (99%) cases. We removed cases where preterm birth may have been medically indicated and excluded births with the following maternal comorbidities, owing to the assumption of different underlying etiologies: pregestational diabetes, gestational diabetes, gestational hypertension, preeclampsia/eclampsia, and chronic hypertension (except for births at 20–23 weeks, with delivery before gestational diabetes is typically diagnosed). This resulted in a final dataset comprising 225,374 births, including 27,913 spontaneous preterm cases and 197,461 term controls (eFigure 1; http://links.lww.com/EE/A91). We further divided preterm birth into three categories based on gestational age: 20–27 weeks (n = 2,307), 28–31 weeks (n = 3,098), and 32–36 weeks (n = 22,508). We considered three categories of preterm births as the etiologic pathway may be different for births delivered at different gestational ages.^21^ Maternal covariates were obtained for each birth, including age (years), race/ethnicity (Hispanic, non-Hispanic Asian, non-Hispanic Black, non-Hispanic White, and other), educational attainment (less than high school, high school, more than high school), and parity (1 or ≥2).

### Exposure assessment

We obtained data on oil and gas wells from the California Geologic Energy Management Division (CalGEM), formerly the Division of Oil, Gas, and Geothermal Resources (DOGGR), as well as Enverus, a private data aggregation service. Data used in this study were obtained in April 2018 and comprised records for 160,256 wells in the San Joaquin Valley, including observations of well type, status, location (latitude and longitude), date of spudding (initial drilling), date of completion (end of preproduction), and the dates production started and ended. We restricted the dataset to wells in the study region that were spudded, completed, or in production between 1 January 1997, and 31 December 2011, to allow for exposures to births in early 1998. We also included wells within 15 km of the boundary of the study region, to allow for exposures to residents living along county borders. A total of 83,559 wells were included in the analytic dataset, including 12,369 wells in preproduction and 71,190 wells in production. Some 3,760 wells were only in the preproduction stage during the study period, 71,190 were only in the production stage, and 8,609 were in both stages. An additional 76,697 wells were neither in preproduction or production during the study period, most of which (77.4%) were plugged and abandoned. We included all wells for which preproduction or production dates were available, due to lack of prior knowledge on the relative hazards associated with different well types. The majority of new and active wells in the analytic dataset were oil and gas wells, including those that use conventional enhanced recovery methods such as steam flooding, cyclic steam injection, and water flooding. For the 12,369 new wells assessed in the current study, the median duration of preproduction (spudding to completion) was 19 days, and 9.3% had long gaps (>100 days) between spudding and completion (eFigure 2; http://links.lww.com/EE/A91). On average, 81.3 ± 75.7 wells were spudded each month, with a range from 6 to 307. The active wells were production for an average of 6,678 days (18.3) years, with a range from 28 to 15,309 days.

Exposure was assessed for each trimester for each birth. The first trimester was defined as gestational weeks 1–13, second trimester as weeks 14–26, and third trimester as week 27 to birth. For wells in preproduction (new wells), we defined an exposure period for each well starting 1 week before spudding and ending 1 week after completion, which allows for oil pad activities around the spud and completion dates. For wells in production (active wells), we defined the exposure period as the interval between production start and end dates provided in the Enverus dataset. We estimated exposure to well sites using an inverse distance-squared weighted index:


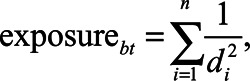


where exposure for birth *b* at trimester *t* is the sum of the inverse-squared Euclidean distance, *d*, to each well, *i*. The exposure assessment was done for all wells, *n*, that were in preproduction or production during trimester *t* within a 10-km radius of the maternal residence. We divided exposed births into exposure tertiles for each trimester of exposure, with group 1 comprising the lowest tertile exposure and group 3 comprising births in the highest exposure tertile. Births without exposure in each trimester comprised a separate unexposed category (tertile “0”). We repeated this procedure to also assess an exposure index only for new wells and only for active wells.

Preterm births delivered during the third trimester have a shorter opportunity for exposure than term births. To account for this potential opportunity bias, we assessed third trimester exposure for only the last 30 days before delivery. We included only births at 32–36 weeks of gestation for this assessment. The majority of births at 20–27 weeks do not have the opportunity to be exposed in the third trimester. Similarly, for the majority of births at 28–31 weeks, the last 30 days of gestation cross the second and third trimesters.

### Statistical analysis

We used logistic regression to estimate the association between exposure to wells and odds of spontaneous preterm birth. We fit models for each gestational age category (20–27, 28–31, and 32–36 weeks) compared with term birth controls (37–41 weeks), with a separate model for each trimester. The analysis included unadjusted models as well as models adjusted for the mother’s age, race/ethnicity, educational attainment, and parity, as well as birth year. We also fit models without the adjustment for birth year.

To test the robustness of findings, we conducted a set of sensitivity analyses. We modified assumptions in the exposure assessment, using radii of (1) 3 km, (2) 5 km, and (3) 15 km of the maternal residence (instead of 10 km); we also used a 10-km radius with (4) inverse distance weighting and (5) inverse distance-square root weighting (instead of inverse distance-squared weighting). For each of these sets of alternative exposure parameters (1–5), we fit crude and adjusted logistic regression models as described above. In a second set of sensitivity analyses, we conducted analyses stratified on maternal race/ethnicity, educational attainment, and birth year. For the birth year stratification, we stratified the analysis into two groups: births from 1998 to 2006 and births from 2007 to 2011. We chose to stratify this way due to a change in the method of estimating gestational age starting in 2007, from last menstrual period to obstetric estimate. For each stratum, we again fit crude and adjusted logistic regression models as described above. We were not able to assess exposure to smoking during pregnancy because we did not have a reliable measure for smoking status. To account for this limitation, we fit adjusted models for the subset of births to Hispanic mothers, a population with low prevalence of smoking.^22^ The prevalence of smoking among Hispanic women during pregnancy was 1.8% in 2016, less than the 7.2% smoking prevalence among all US women.^23^

To examine whether the stage of well development conferred different risks, we conducted a sensitivity analysis confined to the subset of births exposed only to active wells. There were insufficient births to do a similar analysis for births exposed only to new wells. We accounted for potential spatial autocorrelation by fitting mixed-effects models with a random intercept for census tract. To account for potential residual confounding from socioeconomic factors, we fit models adjusted for three additional variables: a categorical variable for mother insurance payer (Medi-Cal, private, uninsured, or other), an indicator for whether prenatal care was initiated before 5 months, and an indicator for whether >20% of families in the census block group were below the poverty level in the 2000 census.

We also conducted a sensitivity analysis that stratified by exposure to traffic-related copollutants: carbon monoxide (CO), nitrogen dioxide (NO_2_), particulate matter with an aerodynamic diameter ≤2.5 µm (PM_2.5_), and particulate matter with an aerodynamic diameter ≤10 µm (PM_10_). For this analysis, we assessed exposure to traffic-related pollutants as described previously by Padula et al.^24^ We obtained traffic exposure data for 85,290 births in the four most populated counties (Fresno, Kern, Stanislaus, and San Joaquin) from 2000 to 2006, which were similar to the whole study population on observed covariates. For this analysis, we assigned each birth an exposure quartile for each of the four traffic-related copollutants. We assigned each birth to either a “low traffic” group if, for all four pollutants and traffic density, the birth was below the highest exposure quartile, or to a “high traffic” group if the birth was in the highest quartile for all five measures.^24^ Among the 85,290 births with traffic data, 32,016 matched either of these criteria, with 29,679 births in the “low traffic” group and 2,337 in the “high traffic” group. These subsets were also similar to the whole sample on observed characteristics. We then fit unadjusted and adjusted logistic regression models as described above, stratified on traffic exposure.

Finally, we conducted a secondary analysis to examine the association between air quality and exposure to drilling sites. For this analysis, we obtained data from the US Environmental Protection Agency (EPA) Air Quality System, which included daily observations of NO_2_, O_3_, PM_10_, and PM_2.5_ from 1998 to 2018 at 290 stations throughout California (eFigure 3; http://links.lww.com/EE/A91). Using statewide data on oil and gas drilling activity, we assessed exposure to drilling sites for each air monitor and for each month from 1998 to 2018, using the same method as described above for maternal residences. For each pollutant, we fit linear models with mean monthly concentration as the dependent variable and exposure tertile as the independent variable. We also fit a model with fixed effects for air basin-month and air basin-year. Air basins are defined by the California Air Resources Board (CARB) with boundaries determined based on similarity of geographic and meteorological features.

### Ethical considerations

The study was approved by the Stanford University Institutional Review Board and the California State Committee for the Protection of Human Subjects.

## Results

The analytic study base included 197,461 term birth controls and 27,913 spontaneous preterm birth cases delivered from 1998 to 2011. Among all births in the analytic dataset, 1.0% (2,307) were delivered at 20–27 weeks of gestational age, 1.4% (3,098) at 28–31 weeks, and the remaining 10.0% (22,508) at 32–36 weeks. The majority of the study population was Hispanic and multiparous, with relatively even distributions of educational attainment (Table 1). Women with preterm deliveries were disproportionately non-Hispanic Black and had disproportionately low educational attainment, compared with women with term births (Table 1). Among all births in the sample, 78,153 were exposed to new or active wells at some point during gestation. The subset with high wells exposure (quantile 3) were comparable to the unexposed births on observe covariates (eTable 1; http://links.lww.com/EE/A91). This observation held when considering exposure separately to new and active wells (eTable 2; http://links.lww.com/EE/A91, eTable 3; http://links.lww.com/EE/A91). Summary statistics for the inverse distance-squared weighted index, organized by tertile for each trimester, are presented in eTable 4; http://links.lww.com/EE/A91, and separately for new wells (eTable 5; http://links.lww.com/EE/A91) and active wells (eTable 6; http://links.lww.com/EE/A91).

**Table 1.**
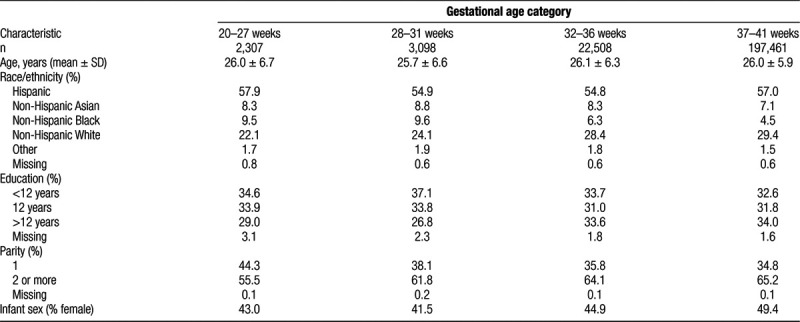
Characteristics of the study population stratified by gestational age category, San Joaquin Valley, CA, 1998–2011

Higher odds of preterm birth were associated with exposure to wells in the highest tertile in the first and second trimesters for births at 20–27 and 28–31 weeks of gestation, with adjusted ORs ranging from 1.08 to 1.14 (Table 2). In unadjusted models, the association between exposure to wells and preterm birth was similar to that observed in adjusted models (eTable 7; http://links.lww.com/EE/A91). The results were consistent in an analysis where we assessed exposure cumulatively throughout gestation (eTable 8; http://links.lww.com/EE/A91). In models adjusted for maternal covariates but not birth year, ORs ranged from 1.10 to 1.35 for exposure in all trimesters and births in all gestational age categories, including births at 32–36 weeks (eTable 9; http://links.lww.com/EE/A91).

**Table 2.**
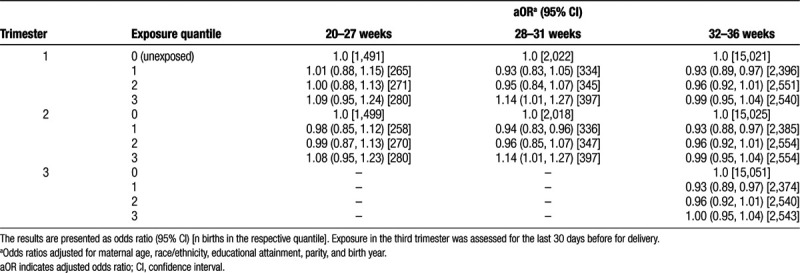
Results for all quantiles of exposure to both new and active wells, compared with unexposed term births, stratified by gestational age category and adjusted for maternal age, race, race/ethnicity, education, and birth year

When we considered exposure separately for new wells (eTable 10; http://links.lww.com/EE/A91) and active wells (eTable 11; http://links.lww.com/EE/A91), the results were consistent with the primary result of an association for exposure in the first and second trimesters confined to births at 20–27 and 28–31 weeks. The majority of exposed births had exposure to both new and active wells. In an analysis confined to the subset of births exposed only to active wells, the association between exposure and preterm birth was confined to births at 20–27 weeks (eTable 12; http://links.lww.com/EE/A91). We also observed an association for the second tertile of exposure for births at 28–31 weeks, but not the highest tertile of exposure.

Using different parameters to assess exposure did not substantially change the observed ORs (eTable 13; http://links.lww.com/EE/A91). In stratified analyses, the observed associations were confined to births to Hispanic and non-Hispanic Black women, and to women with 12 or fewer years of educational attainment (eTable 14; http://links.lww.com/EE/A91). In an analysis stratified by birth year (<2007, ≥2007), we observed ORs from 1.08 to 1.17 for pre-2007, including births at 32–36 weeks. The ORs were attenuated for births from 2007 to 2011, and there was no association for the 32- to 36-week group. In adjusted models for births to Hispanic mothers, a population with low prevalence of smoking, we observed stronger associations than in the entire study population (eTable 15; http://links.lww.com/EE/A91).

Including a random effect for census tract did not substantially change the ORs or 95% confidence intervals (eTable 16; http://links.lww.com/EE/A91). In models that adjusted for additional socioeconomic variables, we similarly did not observe substantial changes in the ORs (eTable 17; http://links.lww.com/EE/A91). When we stratified on traffic-related air pollutant coexposures, the association between exposure to wells and preterm birth (defined in this case as a binary outcome for births at 20–36 weeks) appeared to be persistent for births with low traffic coexposures in the second trimester and for births with high traffic-related coexposures, though these associations were not statistically significant (eTable 18; http://links.lww.com/EE/A91).

In a secondary analysis, ambient air quality monitor-months with high exposure to drilling sites had higher concentrations of PM_10_ and PM_2.5_, compared with unexposed monitor-months (eTable 19; http://links.lww.com/EE/A91). These results were consistent when we added a fixed effects for air basin-month and air-basin year but were attenuated with an additional fixed effect for monitoring station.

## Discussion

We found evidence that exposure to oil and gas well sites in the first and second trimesters is associated with increased odds of spontaneous preterm birth at 20–31 weeks. The association was confined to women who were Hispanic and non-Hispanic Black, and those with 12 or fewer years of educational attainment. Residents near well sites may be exposed to a range of environmental contaminants and stressors, and we were not able to evaluate which factors confer risk. Previous study has found that oil and gas preproduction produces ambient air pollutants, including fine particulate matter, nitrous oxides, volatile organic compounds, ozone, carbon monoxide, and hydrogen sulfide.^1,12–14^ The environmental risks from oil and gas extraction may differ by the stage of preproduction (well pad preparation, road construction, drilling, and completion) or production (different steps in, e.g., cyclic steam injection). We observed an association between preterm birth and exposure to both new and active wells, though due to the correlation in exposure to both new and active wells, we were not able to determine whether exposure to wells in either stage confers more risk. Other potential risk factors include exposure to toxic chemicals used in extraction, but data on the types and toxicity of chemicals used is limited. In oil fields in the South Coast Air Quality Management District, located in Southern California, operators are required to report on-site chemical use.^25^ In oil fields in this area, operators applied 548 chemical additives over a 2-year span, most with unknown toxicity.^25^ In addition, the extent to which human populations have been exposed to oil and gas extraction–related pollutants remains poorly characterized.

In the current study, we found evidence that proximity to wells in preproduction is associated with higher exposure to PM_10_ and PM_2.5_, which supports our hypothesis that proximity to wells in preproduction confers risk. In this analysis, we compared concentrations of ambient air pollutants between exposed and unexposed monitor-months and controlled for seasonal and temporal trends at the air basin level. The results were attenuated, however, when we included a fixed effect for the air monitoring station, though it is unclear which is the correct specification. Further assessments of the air quality impacts of oil and gas operations should consider differences in the fate and transport of ambient air pollutants, account for meteorological factors, consider how the timing of production activities and active wells may affect emissions, and carefully select and specify appropriate statistical models.^26^

There are several possible biological mechanisms for the effect of oil and gas extraction-related environmental exposures on preterm birth. The etiology of preterm birth is suspected to include dysregulated inflammation, which may be a response to infection or oxidative stress associated with air pollution, including particulates and nitrous oxides.^27,28^ The release of proinflammatory mediators, such as cytokines, is associated with exposure to particulates.^4,27^ Psychosocial stress from activities associated with oil and gas preproduction, such as increased noise and traffic, could be an additional contributing factor to preterm birth.^12,29,30^ Recent study has found that exposure to drilling sites in the Marcellus Shale region of central and northwestern Pennsylvania adversely affected mental health, but that increased anxiety and depression did not mediate adverse birth outcomes.^17,31^ There may be additional, unexamined pathways associated with exposure to the chemical additives of unknown toxicity applied in oil and gas extraction.^25^

The current study considers exposure to oil and gas operations in California, where the majority of wells are drilled using conventional methods.^32^ Previous studies of the association between oil and gas development and spontaneous preterm birth have focused on unconventional natural gas extraction in Pennsylvania, Texas, and Colorado. Casey et al.^8^ found an association between preterm birth and exposure to unconventional natural gas wells in the Marcellus Shale of Pennsylvania, with elevated odds ratios for the most exposed tertile of births compared with unexposed. In a quasiexperimental study, Currie et al.^9^ found lower birthweight and poorer overall infant health outcomes for births in areas where unconventional wells were drilled. Whitworth et al.^10^ examined exposure to unconventional natural gas wells in Texas, assessing exposure to wells in the preproduction and production stages. These investigators also found higher odds of preterm birth with exposure to both preproduction and production stages. Null and protective associations between exposure to wells and adverse birth outcomes have been reported by other investigators.^11,33^

This study had several limitations. Exposure was estimated based on proximity and not directly measured. We were not able to account for women who moved between conception and delivery or for exposure at sites other than the residence, which could result in exposure misclassification. This misclassification could bias the results toward the null if moving was not related to preterm birth status, or, conversely, bias in an unpredictable direction if movement was related to preterm birth status. We were not able to account for births to the same mother, which may lead to misestimation of standard errors. The sensitivity analysis for that considered traffic-related coexposures was confined to four counties for which exposure was previously assessed by Padula et al.^24^ We were not able to account other sources of ambient air pollution in the study region. There may be missing observations of well preproduction and production in the data, which could also lead to exposure misclassification. We expect this would produce a bias toward the null. Wells classified as active were assumed to have constant production throughout the study period. If possible, future studies should more carefully consider temporal variations in ambient air pollutant emissions from active wells. In the birth cohort data we used, the method of assessing gestational age shifted in 2007 from last menstrual period to obstetric estimate, which incorporates data from multiple sources and is considered more accurate.^34,35^ With the shift to best obstetric estimate, the defined occurrence of preterm birth is expected to decrease, as the births that may have been misclassified as preterm based on last menstrual period would be more accurately classified as term.^6^ We do not expect misclassification for births at fewer than 31 weeks. Births delivered at 32-36 weeks prior, however, may have been misclassified as preterm if using last menstrual period to assess gestational age. This may explain the result from the analysis stratified on birth year, where the association between exposure and preterm birth was attenuated for late preterm births (32–36 weeks) after 2007. Notably, we still observed an association between exposure to wells and preterm births delivered at fewer than 31 weeks before and after 2007. The current study considers exposure to oil and gas operations in California, where most wells are drilled using conventional methods.

In the future, researchers should consider investigating whether exposure to oil and gas wells may also be associated with medically indicated preterm births, possibly through maternal comorbidities. The findings suggest that future research should consider the reproductive health impacts of both conventional and unconventional drilling. Additional perinatal health outcomes previously associated with exposure to unconventional drilling sites should also be considered, as well as exposure to wells at other stages of development (idle wells and wells in postproduction).

We found an association between exposure to oil and gas well sites and odds of spontaneous preterm birth at 20–31 weeks in the San Joaquin Valley, CA. This study adds to limited evidence that oil and gas extraction activities have adverse impacts on reproductive health.

## Conflict of interest statement

The authors declare that they have no conflicts of interest with regard to the content of this report.

## Acknowledgments

We thank John Oehlert for computational support.

## Supplementary Material


