# Supplementary material for: Oil and gas production and spontaneous preterm birth in the San Joaquin Valley, CA: A case–control study
Source: Environ Epidemiol. 2020 Jun 5;4(4):e099. doi: 10.1097/EE9.0000000000000099 (PMC7423522; doi:10.1097/EE9.0000000000000099)
Supplement: Supplementary file 1 [file ee9-4-e0099-s001.docx]

Supplemental Materials

Oil and gas drilling and risk of spontaneous preterm birth in the San Joaquin Valley, California: A case-control study


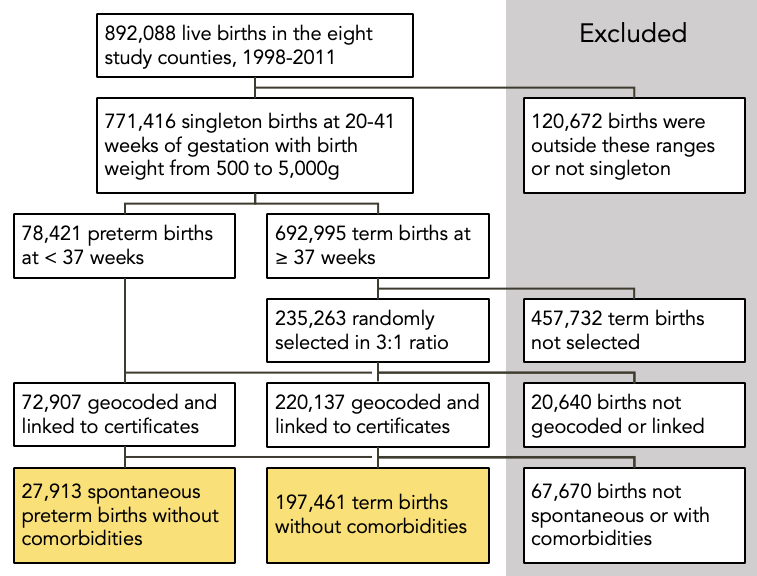


**eFigure 1.** Flow diagram of inclusion and exclusion criteria for the births data.


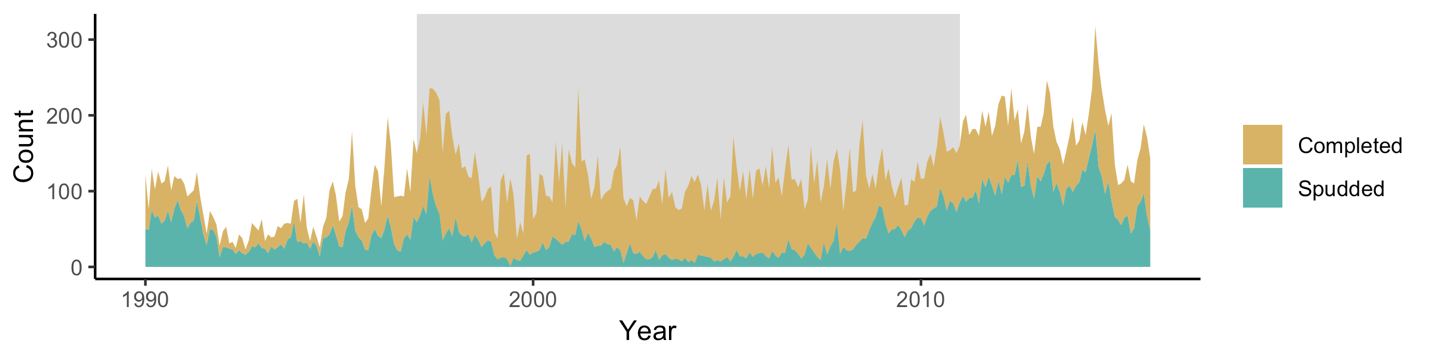


**eFigure 2.** Count of wells spudded and completed by month, California, USA.


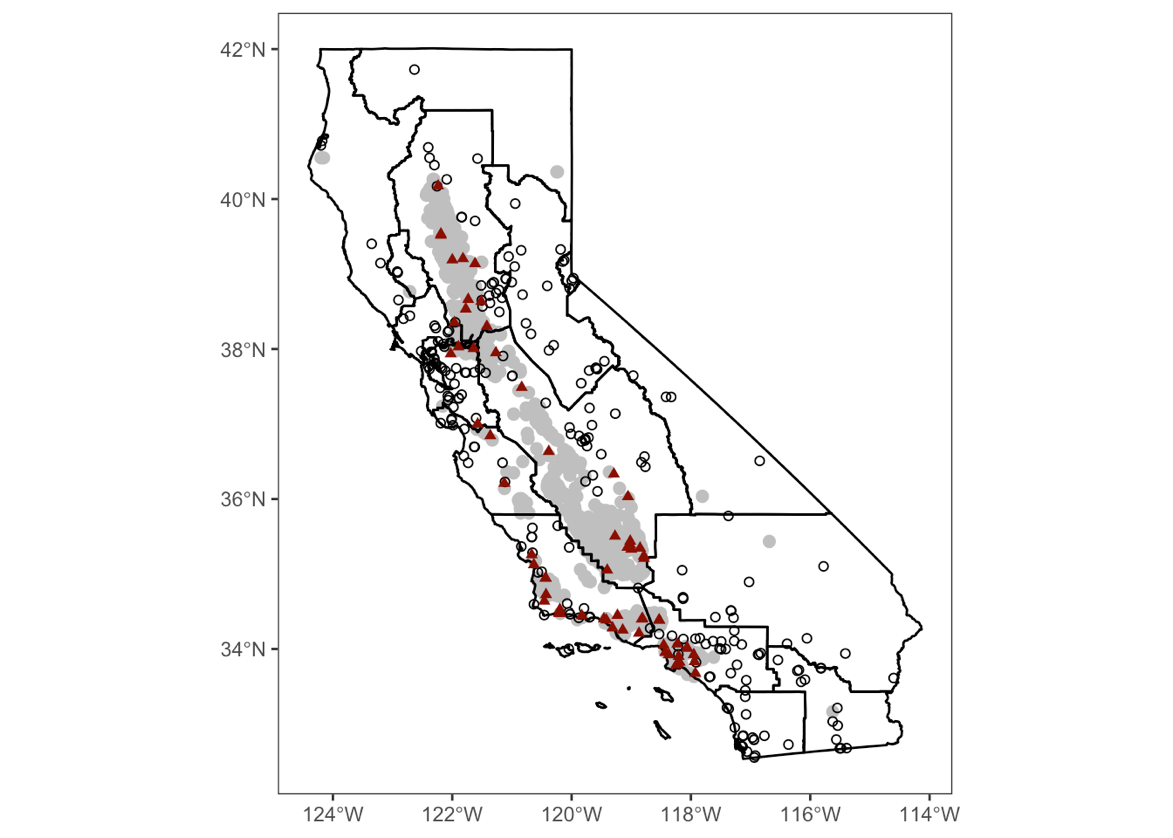


**eFigure 3.** The locations of all U.S. EPA Air Quality System monitoring stations. Stations with at least one month of exposure to oil and gas drilling sites are depicted as red triangles, and stations with no exposure as black circles. The shaded region represents a 10 km buffer around all oil and gas drilling sites, 1998-2018. Air basins, defined by the California Air Resources Board, are outlined in black.

**eTable 1.** Characteristics of the study population stratified by the quantile of exposure to both new and active wells, San Joaquin Valley, California. For this table, exposure was assessed for the entire duration of gestation rather than by trimester.

|  |  | Exposure | quantile |  |
| --- | --- | --- | --- | --- |
| Covariates | 0 (none) | 1 | 2 | 3 |
| n | 147,221 | 26,051 | 26,051 | 26,051 |
| Gestational age (%) |  |  |  |  |
| 20-27 weeks | 1.0 | 1.0 | 1.0 | 1.1 |
| 28-31 weeks | 1.4 | 1.3 | 1.4 | 1.6 |
| 32-36 weeks | 10.1 | 9.3 | 10.0 | 10.0 |
| 37-41 weeks (term) | 87.5 | 88.4 | 87.6 | 87.4 |
| Age, years (mean ± SD) | 26.1 ± 6.0 | 26.5 ± 6.1 | 25.9 ± 6.0 | 25.7 ± 5.9 |
| Race/ethnicity (%) |  |  |  |  |
| Hispanic | 56.6 | 52.2 | 64.4 | 54.9 |
| Non-Hispanic Asian | 7.3 | 11.2 | 7.4 | 2.4 |
| Non-Hispanic Black | 4.1 | 5.6 | 6.4 | 6.2 |
| Non-Hispanic White | 29.9 | 28.9 | 20.0 | 34.1 |
| Other | 1.6 | 1.5 | 1.4 | 1.6 |
| Missing | 0.5 | 0.5 | 0.5 | 0.8 |
| Education (%) |  |  |  |  |
| < 12 years | 33.0 | 30.9 | 36.4 | 30.1 |
| 12 years | 31.0 | 31.8 | 33.5 | 33.9 |
| > 12 years | 34.9 | 35.8 | 27.6 | 31.5 |
| Missing | 1.1 | 1.5 | 2.5 | 4.5 |
| Parity (%) |  |  |  |  |
| 1 | 35.0 | 34.8 | 33.8 | 36.3 |
| 2 or more | 64.9 | 65.1 | 66.1 | 63.5 |
| Missing | 0.1 | 0 | 0.1 | 0.2 |
| Infant sex (% female) | 48.6 | 48.9 | 48.9 | 49.1 |

**eTable 2.** Characteristics of the study population stratified by the quantile of exposure to new wells, San Joaquin Valley, California. For this table, exposure was assessed for the entire duration of gestation rather than by trimester.

|  |  | Exposure | quantile |  |
| --- | --- | --- | --- | --- |
| Covariates | 0 (none) | 1 | 2 | 3 |
| n | 181,354 | 14,674 | 14,673 | 14,673 |
| Gestational age (%) |  |  |  |  |
| 20-27 weeks | 1.0 | 1.0 | 1.0 | 1.0 |
| 28-31 weeks | 1.4 | 1.3 | 1.4 | 1.5 |
| 32-36 weeks | 10.0 | 9.5 | 10.0 | 10.3 |
| 37-41 weeks (term) | 87.6 | 88.2 | 87.6 | 87.2 |
| Age, years (mean ± SD) | 26.1 ± 5.97 | 26.2 ± 6.02 | 25.7 ± 6.09 | 25.5 ± 5.89 |
| Race/ethnicity (%) |  |  |  |  |
| Hispanic | 56.4 | 55.2 | 64.5 | 55.5 |
| Non-Hispanic Asian | 7.4 | 8.8 | 6.5 | 3.4 |
| Non-Hispanic Black | 4.3 | 6.6 | 6.9 | 6.2 |
| Non-Hispanic White | 29.8 | 27.5 | 19.8 | 32.4 |
| Other | 1.5 | 1.4 | 1.5 | 1.8 |
| Missing | 0.5 | 0.5 | 0.6 | 0.8 |
| Education (%) |  |  |  |  |
| < 12 years | 32.6 | 31.0 | 37.6 | 32.3 |
| 12 years | 31.3 | 33.2 | 32.5 | 35.5 |
| > 12 years | 34.9 | 33.4 | 26.7 | 27.8 |
| Missing | 1.3 | 2.4 | 3.3 | 4.4 |
| Parity (%) |  |  |  |  |
| 1 | 34.9 | 35.4 | 34.9 | 35.4 |
| 2 or more | 65.0 | 64.5 | 65.1 | 64.5 |
| Missing | 0.1 | 0.1 | 0.1 | 0.1 |
| Infant sex (% female) | 48.6 | 49.3 | 48.9 | 49.2 |

**eTable 3.** Characteristics of the study population stratified by quantile of exposure to active wells, San Joaquin Valley, California. For this table, exposure was assessed for the entire duration of gestation rather than by trimester.

|  |  | Exposure | quantile |  |
| --- | --- | --- | --- | --- |
| Covariates | 0 (none) | 1 | 2 | 3 |
| n | 151,858 | 24,506 | 24,505 | 24,505 |
| Gestational age (%) |  |  |  |  |
| 20-27 weeks | 1.0 | 1.1 | 1.0 | 1.1 |
| 28-31 weeks | 1.4 | 1.3 | 1.3 | 1.6 |
| 32-36 weeks | 10.1 | 9.6 | 9.9 | 10.0 |
| 37-41 weeks (term) | 87.6 | 88.0 | 87.8 | 87.3 |
| Age, years (mean ± SD) | 26.1 ± 6.0 | 26.5 ± 6.0 | 25.9 ± 6.0 | 25.6 ± 5.9 |
| Race/ethnicity (%) |  |  |  |  |
| Hispanic | 56.9 | 50.0 | 64.1 | 55.3 |
| Non-Hispanic Asian | 7.4 | 11.1 | 7.0 | 2.2 |
| Non-Hispanic Black | 4.0 | 6.5 | 6.2 | 6.1 |
| Non-Hispanic White | 29.6 | 30.4 | 20.6 | 34.0 |
| Other | 1.6 | 1.5 | 1.5 | 1.6 |
| Missing | 0.5 | 0.5 | 0.6 | 0.8 |
| Education (%) |  |  |  |  |
| < 12 years | 33.2 | 30.2 | 35.1 | 30.6 |
| 12 years | 31.1 | 31.6 | 33.6 | 34.0 |
| > 12 years | 34.7 | 36.7 | 28.6 | 30.8 |
| Missing | 1.1 | 1.5 | 2.7 | 4.5 |
| Parity (%) |  |  |  |  |
| 1 | 35.0 | 34.7 | 34.2 | 36.2 |
| 2 or more | 64.9 | 65.3 | 65.8 | 63.7 |
| Missing | 0.1 | 0 | 0.1 | 0.2 |
| Infant sex (% female) | 48.7 | 48.8 | 48.8 | 49.0 |

**eTable 4.** Summary statistics for inverse distance-squared weighted (IDW) indices for exposure to both new and active wells, organized by trimester of exposure. The values are for each exposure tertile. Exposed births were divided into quantiles; quantile “0” comprises unexposed births. An index of 1 is equivalent to having one well located 1 km away from the maternal residence, or ten wells located 10 km away.

| Trimester | Quantile | Mean ± SD | Range |
| --- | --- | --- | --- |
| 1 | 0 (unexposed) | 0 | 0 |
|  | 1 | 0.08 ± 0.06 | 0.01, 0.23 |
|  | 2 | 2.97 ± 3.23 | 0.23, 11.59 |
|  | 3 | 139.9 ± 169.8 | 11.6, 3,326.9 |
| 2 | 0 | 0 | 0 |
|  | 1 | 0.08 ± 0.06 | 0.01, 0.23 |
|  | 2 | 2.97 ± 3.21 | 0.23, 11.53 |
|  | 3 | 140.0 ± 170.1 | 11.5, 3,327.1 |
| 3 | 0 | 0 | 0 |
|  | 1 | 0.08 ± 0.06 | 0.01, 0.23 |
|  | 2 | 2.95 ± 3.20 | 0.23, 11.46 |
|  | 3 | 140.0 ± 170.6 | 11.5, 3,326.5 |

**eTable 5.** Summary statistics for inverse distance-squared weighted (IDW) indices for exposure to new wells, organized by trimester of exposure. The values are for each exposure tertile. Exposed births were divided into tertiles; tertile “0” comprises unexposed births.

| Trimester | Quantile | Mean ± SD | Range |
| --- | --- | --- | --- |
| 1 | 0 (unexposed) | 0 | 0 |
|  | 1 | 0.02 ± 0.01 | 0.01, 0.03 |
|  | 2 | 0.07 ± 0.03 | 0.03, 0.14 |
|  | 3 | 0.86 ± 1.54 | 0.14, 58.56 |
| 2 | 0 | 0 | 0 |
|  | 1 | 0.02 ± 0.01 | 0.01, 0.03 |
|  | 2 | 0.07 ± 0.03 | 0.03, 0.15 |
|  | 3 | 0.93 ± 1.72 | 0.15, 84.3 |
| 3 | 0 | 0 | 0 |
|  | 1 | 0.02 ± 0.01 | 0.01, 0.03 |
|  | 2 | 0.07 ± 0.03 | 0.03, 0.14 |
|  | 3 | 0.91 ± 1.66 | 0.15, 76.68 |

**eTable 6.** Summary statistics for inverse distance-squared weighted (IDW) indices for exposure to active wells, organized by trimester of exposure. The values are for each exposure tertile. Exposed births were divided into tertiles; tertile “0” comprises unexposed births.

| Trimester | Quantile | Mean ± SD | Range |
| --- | --- | --- | --- |
| 1 | 0 (unexposed) | 0 | 0 |
|  | 1 | 0.09 ± 0.07 | 0.01, 0.28 |
|  | 2 | 3.63 ± 3.71 | 0.28, 13.00 |
|  | 3 | 145.4 ± 171.0 | 13.0, 3,326.9 |
| 2 | 0 | 0 | 0 |
|  | 1 | 0.09 ± 0.07 | 0.01, 0.28 |
|  | 2 | 3.62 ± 3.69 | 0.28, 12.94 |
|  | 3 | 145.5 ± 171.3 | 12.9, 3,327.1 |
| 3 | 0 | 0 | 0 |
|  | 1 | 0.09 ± 0.07 | 0.01, 0.29 |
|  | 2 | 3.61 ± 3.68 | 0.29, 12.88 |
|  | 3 | 145.5 ± 171.7 | 12.9, 3,326.5 |

**eTable 7.** Unadjusted results for all quantiles of exposure to both new and active wells compared with unexposed births, stratified by gestational age category. The results are presented as unadjusted odds ratio (95% confidence interval). Exposure in the third trimester was assessed for the last 30 days prior to delivery.

|  |  |  | OR (95% CI) |  |
| --- | --- | --- | --- | --- |
| Trimester | Exposure Tertile | 20-27 weeks | 28-31 weeks | 32-36 weeks |
| 1 | 0 (unexposed) | 1.0 | 1.0 | 1.0 |
|  | 1 | 1.03 (0.90, 1.18) | 0.96 (0.85, 1.08) | 0.93 (0.89, 0.97) |
|  | 2 | 1.07 (0.93, 1.21) | 1.00 (0.89, 1.12) | 1.00 (0.95, 1.04) |
|  | 3 | 1.10 (0.97, 1.25) | 1.15 (1.03, 1.28) | 0.99 (0.95, 1.04) |
| 2 | 0 | 1.0 | 1.0 | 1.0 |
|  | 1 | 1.00 (0.87, 1.14) | 0.97 (0.86, 1.08) | 0.92 (0.88, 0.96) |
|  | 2 | 1.05 (0.92, 1.20) | 1.01 (0.90, 1.13) | 1.00 (0.95, 1.04) |
|  | 3 | 1.10 (0.96, 1.24) | 1.15 (1.03, 1.29) | 0.99 (0.95, 1.04) |
| 3 | 0 | – | – | 1.0 |
|  | 1 | – | – | 0.92 (0.88, 0.97) |
|  | 2 | – | – | 0.99 (0.95, 1.04) |
|  | 3 | – | – | 1.00 (0.95, 1.04) |

**eTable 8.** Results of logistic regression models with exposure assessed cumulatively throughout entire duration of pregnancy. Presented as adjusted odds ratios (95% confidence intervals) [*n* births in the respective quantile].

|  |  | aOR (95% CI) |  |
| --- | --- | --- | --- |
| Exposure Tertile | 20-27 weeks | 28-31 weeks | 32-36 weeks |
| 0 | 1.0 [1,490] | 1.0 [2,004] | 1.0 [14,891] |
| 1 | 0.95 (0.83, 1.08) [258] | 0.91 (0.81, 1.03) [336] | 0.91 (0.87, 0.96) [2,419] |
| 2 | 0.98 (0.85, 1.11) [273] | 0.94 (0.84, 1.06) [352] | 0.96 (0.91, 1.00) [2,596] |
| 3 | 1.08 (0.94, 1.22) [286] | 1.13 (1.01, 1.26) [406] | 0.99 (0.95, 1.04) [2,602] |

^a^ Odds ratios adjusted for maternal age, race/ethnicity, educational attainment, parity, and birth year.

**eTable 9.** Results adjusted for maternal covariates but not for birth year. Presented as adjusted odds ratio (95% confidence interval). Exposure in the third trimester was assessed for the last 30 days prior to delivery.

|  |  |  | aOR (95% CI) |  |
| --- | --- | --- | --- | --- |
| Trimester | Exposure Tertile | 20-27 weeks | 28-31 weeks | 32-36 weeks |
| 1 | 0 (unexposed) | 1.0 | 1.0 | 1.0 |
|  | 1 | 1.15 (0.95, 1.36) | 1.07 (0.91, 1.25) | 1.03 (0.97, 1.10) |
|  | 2 | 1.02 (0.84, 1.23) | 1.10 (0.94, 1.29) | 1.04 (0.97, 1.10) |
|  | 3 | 1.21 (1.00, 1.44) | 1.35 (1.16, 1.56) | 1.10 (1.03, 1.17) |
| 2 | 0 | 1.0 | 1.0 | 1.0 |
|  | 1 | 0.95 (0.78, 1.14) | 1.00 (0.85, 1.18) | 0.98 (0.92, 1.05) |
|  | 2 | 1.00 (0.82, 1.20) | 1.13 (0.97., 1.32) | 1.04 (0.97, 1.10) |
|  | 3 | 1.14 (0.95, 1.37) | 1.24 (1.06, 1.44) | 1.12 (1.05, 1.19) |
| 3 | 0 | – | – | 1.0 |
|  | 1 | – | – | 1.02 (0.96, 1.08) |
|  | 2 | – | – | 1.09 (1.03, 1.16) |
|  | 3 | – | – | 1.12 (1.05, 1.18) |

^a^ Odds ratios adjusted for maternal age, race/ethnicity, educational attainment, and parity.

**eTable 10.** Results for all quantiles of exposure to drilling sites (new wells) compared with unexposed births, stratified by gestational age category. The results are presented as adjusted odds ratio (95% confidence interval) [*n* births in the respective quantile]. Exposure in the third trimester was assessed for the last 30 days prior to delivery.

|  |  |  | aOR (95% CI) |  |
| --- | --- | --- | --- | --- |
| Trimester | Exposure Tertile | 20-27 weeks | 28-31 weeks | 32-36 weeks |
| 1 | 0 (unexposed) | 1.0  [1,906] | 1.0 [2,546] | 1.0 [18,896] |
|  | 1 | 1.14 (0.95, 1.35) [138] | 1.06 (0.90, 1.24) [169] | 1.01 (0.95, 1.08) [1,190] |
|  | 2 | 1.02 (0.84, 1.22) [128] | 1.10 (0.93, 1.28) [174] | 1.03 (0.97, 1.10) [1,182] |
|  | 3 | 1.12 (0.93, 1.34) [135] | 1.31 (1.13, 1.52) [209] | 0.99 (0.93, 1.06) [1,240] |
| 2 | 0 | 1.0 [1,936] | 1.0 [2,564] | 1.0 [18,911] |
|  | 1 | 0.95 (0.78, 1.14) [120] | 1.00 (0.84, 1.17) [159] | 0.97 (0.91, 1.03) [1,147] |
|  | 2 | 1.00 (0.93, 1.20) [121] | 1.12 (0.96, 1.31) [180] | 1.04 (0.97, 1.10) [1,192] |
|  | 3 | 1.06 (0.88, 1.27) [130] | 1.20 (1.03, 1.40) [195] | 1.00 (0.94, 1.07) [1,258] |
| 3 | 0 | – | – | 1.0 [19,224] |
|  | 1 | – | – | 1.02 (0.95, 1.09) [1,076] |
|  | 2 | – | – | 1.10 (1.03, 1.17) [1,102] |
|  | 3 | – | – | 0.98 (0.91, 1.05) [1,106] |

^a^ Odds ratios adjusted for maternal age, race/ethnicity, educational attainment, parity, and birth year.

**eTable 11.** Results for all quantiles of exposure to wells in production during the study period (active wells) compared with unexposed births, stratified by gestational age category. The results are presented as adjusted odds ratio (95% confidence interval) [*n* births in the respective quantile]. Exposure in the third trimester was assessed for the last 30 days prior to delivery.

|  |  |  | aOR (95% CI) |  |
| --- | --- | --- | --- | --- |
| Trimester | Exposure Tertile | 20-27 weeks | 28-31 weeks | 32-36 weeks |
| 1 | 0 (unexposed) | 1.0  [1,525] | 1.0 [2,071] | 1.0 [15,312] |
|  | 1 | 1.08 (0.94, 1.23) [267] | 0.91 (0.80, 1.03) [311] | 0.96 (0.91, 1.00) [2,347] |
|  | 2 | 0.96 (0.83, 1.10) [247] | 0.94 (0.83, 1.06) [326] | 0.96 (0.91, 1.00) [2,415] |
|  | 3 | 1.10 (0.96, 1.26) [268] | 1.17 (1.04, 1.31) [390] | 1.00 (0.96, 1.05) [2,434] |
| 2 | 0 | 1.0 [1,528] | 1.0 [2,065] | 1.0 [15,312] |
|  | 1 | 1.05 (0.92, 1.20) [261] | 0.93 (0.83, 1.05) [318] | 0.96 (0.92, 1.01) [2,350] |
|  | 2 | 0.97 (0.84, 1.11) [251] | 0.94 (0.83, 1.06) [325] | 0.96 (0.91, 1.00) [2,409] |
|  | 3 | 1.09 (0.95, 1.25) [267] | 1.17 (1.04, 1.31) [390] | 1.00 (0.96, 1.05) [2,437] |
| 3 | 0 | – | – | 1.0 [15,329] |
|  | 1 | – | – | 0.96 (0.92, 1.01) [2,345] |
|  | 2 | – | – | 0.95 (0.91, 1.00) [2,397] |
|  | 3 | – | – | 1.01 (0.96, 1.05) [2,437] |

^a^ Odds ratios adjusted for maternal age, race/ethnicity, educational attainment, parity, and birth year.

**eTable 12.** Results from a sensitivity analysis for the subset of births exposed only to active wells compared to unexposed term births. The results are presented as adjusted odds ratios (95% confidence intervals) [*n* births in the respective quantile]. Exposure in the third trimester was assessed for the last 30 days prior to delivery.

|  |  |  | aOR (95% CI) |  |
| --- | --- | --- | --- | --- |
| Trimester | Exposure Tertile | 20-27 weeks | 28-31 weeks | 32-36 weeks |
| 1 | 0 (unexposed) | 1.0 [1,490] | 1.0 [2,013] | 1.0 [14,970] |
|  | 1 | 1.08 (0.91, 1.26) [172] | 0.89 (0.76, 1.03) [190] | 0.95 (0.90, 1.01) [1,519] |
|  | 2 | 1.12 (0.95, 1.32) [158] | 1.12 (0.96, 1.29) [216] | 0.93 (0.88, 0.99) [1,297] |
|  | 3 | 1.20 (0.88, 1.59) [50] | 0.96 (0.72, 1.26) [58] | 1.01 (0.91, 1.12) [396] |
| 2 | 0 | 1.0 [1,493] | 1.0 [2,010] | 1.0 [14,975] |
|  | 1 | 1.05 (0.89, 1.24) [168] | 0.91 (0.78, 1.05) [193] | 0.96 (0.91, 1.02) [1,518] |
|  | 2 | 1.13 (0.95, 1.33) [159] | 1.12 (0.97, 1.29) [216] | 0.93 (0.87, 0.99) [1,291] |
|  | 3 | 1.20 (0.88, 1.59) [50] | 0.96 (0.72, 1.26) [58] | 1.02 (0.91, 1.13) [398] |
| 3 | 0 | – | – | 1.0 [15,128] |
|  | 1 | – | – | 0.96 (0.91, 1.02) [1,506] |
|  | 2 | – | – | 0.93 (0.87, 0.99) [1,286] |
|  | 3 | – | – | 1.03 (0.92, 1.15) [393] |

^a^ Odds ratios adjusted for maternal age, race/ethnicity, educational attainment, parity, and birth year.

**eTable 13.** Results from sensitivity analysis with alternative exposure assessment assumptions. For these analyses, we assessed exposure using different parameters. We assessed exposure to wells within (a) 3, (b) 5, and (c) 15 km of the maternal residence using inverse distance-squared weighting, and to wells within 10 km with (d) inverse distance weighting and (e) inverse distance-square root weighting. Results are presented as adjusted odds ratios (95% confidence intervals) [*n* births in the respective quantile]. For assessment (a), exposure is assigned as a binary indicator (exposed/unexposed) rather than divided into tertiles, because the range of IDW indices were comparable to that of high tertile births in other assessments.

|  |  |  | aOR (95% CI) |  |
| --- | --- | --- | --- | --- |
| Exposure Parameters | Trimester | 20-27 weeks | 28-31 weeks | 32-36 weeks |
|  | 1 | 1.09 (0.95, 1.24) | 1.14 (1.01, 1.27) | 0.99 (0.95, 1.04) |
| 10 km radius, (1/d^2^) | 2 | 1.08 (0.95, 1.23) | 1.14 (1.01, 1.27) | 0.99 (0.95, 1.04) |
| (primary result) | 3 | – | – | 1.00 (0.95, 1.04) |
|  | 1 | 1.07 (0.95, 1.21) [326] | 1.05 (0.94, 1.17) [435] | 0.95 (0.91, 0.99) [2,882] |
| (a) 3 km radius, (1/d^2^) | 2 | 1.07 (0.94, 1.20) [324] | 1.05 (0.94, 1.16) [434] | 0.95 (0.91, 1.00) [2,881] |
|  | 3 | – | – | 0.97 (0.92, 1.00) [2,848] |
|  | 1 | 1.02 (0.86, 1.20) [162] | 1.14 (0.99, 1.31) [245] | 0.98 (0.92, 1.03) [1,569] |
| (b) 5 km radius, (1/d^2^) | 2 | 1.01 (0.85, 1.19) [161] | 1.14 (0.99, 1.30) [244] | 0.98 (0.92, 1.03) [1,564] |
|  | 3 | – | – | 0.98 (0.92, 1.03) [1,558] |
|  | 1 | 1.08 (0.95, 1.21) [373] | 1.16 (1.04, 1.28) [529] | 0.97 (0.93, 1.02) [3,302] |
| (c) 15 km radius, (1/d^2^) | 2 | 1.07 (0.95, 1.21) [373] | 1.16 (1.05, 1.28) [532] | 0.97 (0.93, 1.01) [3,312] |
|  | 3 | – | – | 0.97 (0.93, 1.02) [3,295] |
|  | 1 | 1.09 (0.95, 1.24) [279] | 1.20 (1.07, 1.34) [416] | 1.01 (0.96, 1.05) [2,560] |
| (d) 10 km radius, (1/d) | 2 | 1.08 (0.94, 1.23) [279] | 1.20 (1.07, 1.34) [417] | 1.01 (0.96, 1.05) [2,559] |
|  | 3 | – | – | 1.01 (0.96, 1.06) [2,545] |
|  | 1 | 1.10 (0.96, 1.25) [282] | 1.19 (1.06, 1.32) [410] | 1.00 (0.96, 1.05) [2,556] |
| (e) 10 km radius, (1/d^½^) | 2 | 1.08 (0.95, 1.23) [280] | 1.19 (1.06, 1.32) [410] | 1.00 (0.96, 1.05) [2,561] |
|  | 3 | – | – | 1.00 (0.96, 1.05) [2,540] |

^a^ Odds ratios adjusted for maternal age, race/ethnicity, educational attainment, parity, and birth year.

**eTable 14.** Results from analyses stratified by (a) maternal race/ethnicity, (b) maternal education, and (c) birth year. We fit unadjusted logistic regression models for each stratum, and each trimester and gestational age category within the stratum. For the stratification on maternal race/ethnicity, we omitted the strata for Asian and other due to insufficient sample size among the subset of births with high exposure. Presented as unadjusted OR (95% CI) [*n* births in high exposure tercile].

|  |  |  |  | Gestational age |  |
| --- | --- | --- | --- | --- | --- |
|  | Stratum | Trimester | 20-27 weeks | 28-31 weeks | 32-36 weeks |
|  |  | 1 | 1.15 (0.97, 1.36) [165] | 1.22 (1.05, 1.41) [224] | 1.05 (0.99, 1.11) [1,412] |
|  | Hispanic | 2 | 1.15 (0.97, 1.35) [165] | 1.22 (1.05, 1.41) [224] | 1.05 (0.99, 1.11) [1,413] |
|  |  | 3 | – | – | 1.05 (0.99, 1.11) [1,406] |
| (a) Maternal |  | 1 | 1.23 (0.82, 1.79) [34] | 1.18 (0.84, 1.63) [48] | 1.13 (0.96, 1.33) [224] |
| race/ethnicity | Non-Hispanic Black | 2 | 1.22 (0.82, 1.79) [34] | 1.18 (0.84, 1.62) [48] | 1.12 (0.96, 1.32) [224] |
|  |  | 3 | – | – | 1.13 (0.96, 1.33) [223] |
|  |  | 1 | 1.00 (0.76, 1.29) [67] | 1.02 (0.82, 1.26) [101] | 0.88 (0.81, 0.95) [766] |
|  | Non-Hispanic White | 2 | 0.99 (0.76, 1.28) [67] | 1.03 (0.82, 1.27) [101] | 0.88 (0.82, 0.95) [769] |
|  |  | 3 | – | – | 0.88 (0.82, 0.96) [764] |
|  |  | 1 | 1.27 (1.02, 1.57) [101] | 1.42 (1.19, 1.68) [162] | 1.16 (1.07, 1.25) [876] |
|  | < 12 years | 2 | 1.27 (1.02, 1.56) [101] | 1.41 (1.18, 1.67) [162] | 1.16 (1.07, 1.25) [880] |
|  |  | 3 | – | – | 1.16 (1.07, 1.25) [877] |
| (b) Maternal |  | 1 | 1.06 (0.85, 1.31) [99] | 1.05 (0.86, 1.26) [132] | 0.99 (0.91, 1.07) [843] |
| educational | 12 years | 2 | 1.04 (0.93, 1.29) [99] | 1.06 (0.87, 1.27) [132] | 0.99 (0.91, 1.07) [843] |
| attainment |  | 3 | – | – | 0.99 (0.91, 1.07) [839] |
|  |  | 1 | 0.95 (0.73, 1.23) [65] | 0.93 (0.73, 1.17) [82] | 0.85 (0.78, 0.92) [700] |
|  | > 12 years | 2 | 0.95 (0.72, 1.22) [65] | 0.93 (0.73, 1.17) [82] | 0.85 (0.78, 0.92) [703] |
|  |  | 3 | – | – | 0.85 (0.78, 0.92) [695] |
|  |  |  |  |  |  |
|  |  | 1 | 1.14 (0.96, 1.98) [170] | 1.17 (1.01, 1.93) [250] | 1.08 (1.02, 1.22) [1,462] |
|  | 1998-2006 | 2 | 1.13 (0.95, 1.98) [170] | 1.17 (1.01, 1.93) [250] | 1.09 (1.02, 1.22) [1,466] |
| (c) Birth year |  | 3 | – | – | 1.10 (1.04, 1.17) [1,462] |
|  |  | 1 | 1.03 (0.83, 1.26) [110] | 1.08 (0.90, 1.08) [147] | 0.88 (0.82, 1.13) [1,077] |
|  | 2007-2011 | 2 | 1.02 (0.82, 1.26) [110] | 1.08 (0.90, 1.08) [147] | 0.88 (0.82, 1.13) [1,078] |
|  |  | 3 | – | – | 0.87 (0.81, 0.93) [1,068] |

**eTable 15.** Results from an analysis on the subset of births to Hispanic mothers. Results are presented as adjusted odds ratios (95% confidence intervals). This analysis compares the most exposed births (tertile 3) with unexposed births with exposure assessed to new and active wells.

|  |  | aOR (95% CI) |  |
| --- | --- | --- | --- |
| Trimester | 20-27 weeks | 28-31 weeks | 32-36 weeks |
| 1 | 1.14 (0.96, 1.35) | 1.23 (1.06, 1.42) | 1.04 (0.98, 1.10) |
| 2 | 1.13 (0.95, 1.34) | 1.23 (1.06, 1.42) | 1.04 (0.98, 1.10) |
| 3 | – | – | 1.04 (0.98, 1.11) |

^a^ Odds ratios adjusted for maternal age, educational attainment, parity, and birth year.

**eTable 16.** Results from a sensitivity analysis that accounts for potential spatial autocorrelation in the exposure. Results are presented as odds ratios (95% confidence intervals) for the association between high exposure to well sites and preterm birth, adjusted for all factors included in the primary analysis with the addition of a random effect for census tract. This analysis compares the most exposed births (tertile 3) with unexposed births.

|  |  | aOR (95% CI) |  |
| --- | --- | --- | --- |
| Trimester | 20-27 weeks | 28-31 weeks | 32-36 weeks |
| 1 | 1.07 (0.92 1.25) | 1.13 (1.00, 1.28) | 0.97 (0.91, 1.03) |
| 2 | 1.07 (0.92, 1.24) | 1.13 (1.00, 1.28) | 0.97 (0.91, 1.03) |
| 3 | – | – | 0.97 (0.92, 1.04) |

^a^ Odds ratios adjusted for maternal age, race/ethnicity, educational attainment, parity, and birth year.

**eTable 17.** Results from a sensitivity analysis adjusting for additional factors related to socioeconomic status. Odds ratios (95% confidence intervals) for the association between high exposure to well sites and preterm birth, adjusted for all factors included in the primary analysis as well as mother insurance payer, an indicator whether prenatal care was initiated before 5 months of gestation, and an indicator for whether the more than 20% of families were below the poverty level in the U.S. Census block group. This analysis compares the most exposed births (tertile 3) with unexposed births.

|  |  | aOR (95% CI) |  |
| --- | --- | --- | --- |
| Trimester | 20-27 weeks | 28-31 weeks | 32-36 weeks |
| 1 | 1.14 (0.99, 1.31) | 1.15 (1.02, 1.29) | 0.99 (0.94, 1.04) |
| 2 | 1.14 (0.99, 1.30) | 1.15 (1.02, 1.29) | 0.99 (0.94, 1.04) |
| 3 | – | – | 1.00 (0.95, 1.04) |

^a^ Odds ratios adjusted for maternal age, race/ethnicity, educational attainment, parity, birth year, mother insurance payer, an indicator whether prenatal care was initiated before 5 months, and an indicator for whether >20% of families in the neighborhood were below the poverty level.

**eTable 18.** Results from a sensitivity analysis stratified on exposure to traffic-related copollutants. Births with low exposure to traffic-related copollutants (n = 29,679) were defined as those in the lowest three quartiles for exposures to all four measured copollutants and traffic density. Births with high exposure (n = 2,337) were in the highest quartile for all five measures. The final combined column includes results full subset of births for which traffic exposure was assessed (n = 32,016). Results are presented as adjusted odds ratios (95% confidence intervals). This analysis compares the most exposed births (tertile 3) with unexposed births.

|  |  | aOR (95% CI) |  |
| --- | --- | --- | --- |
| Trimester | Low traffic exposure | High traffic exposure | Combined low and high traffic exposure |
| 1 | 0.99 (0.73, 1.32) | 1.22 (0.80, 1.83) | 1.11 (0.87, 1.40) |
| 2 | 1.09 (0.80, 1.43) | 1.13 (0.74, 1.69) | 1.15 (0.90, 1.43) |
| 3 | 1.00 (0.98, 1.03) | 1.11 (0.66, 1.79) | 1.02 (0.79, 1.30) |

^a^ Odds ratios adjusted for maternal age, race/ethnicity, educational attainment, parity, and birth year.

**eTable 19.** Results from the secondary analysis of the effect of exposure to drilling sites on air quality, comparing the most exposed station-months (i.e., those in the highest exposure tertile) to unexposed station-months. We fit separate models for each pollutant. Model 1 is an unadjusted linear regression model, model 2 includes fixed effects for air basin-month and air basin-year, and model 3 includes an additional fixed effect for air monitoring station. This analysis uses data from U.S. EPA air pollution monitoring stations. For each station, we assessed exposure as described for the births, and the same inverse distance-squared weighted index cutoffs were used to define the exposure quantiles. Point estimates may be interpreted as the change in the observed pollutant concentration for among monitor-months in the highest exposure tertile compared to unexposed monitor-months.

| Pollutant | Model 1^a^ | Model 2^b^ | Model 3^c^ |
| --- | --- | --- | --- |
| NO_2_ (ppb) | 5.92*** | -0.094** | -0.83** |
| O_3_ (ppm) | <0.001*** | <0.001** | <0.001 |
| PM_10_ (µg/m^3^) | 8.99*** | 3.76*** | 0.85^•^ |
| PM_2.5_ (µg/m^3^) | 4.00*** | 1.30*** | 0.43 |

^a^ Linear regression model with no fixed effects. ^b^ Linear regression model with fixed effects for air basin-month and air basin-year. ^c^ Linear regression model with fixed effects for air basin-month, air basin-year, and air monitoring station.

*** p < 0.001, ** p < 0.01, * p < 0.05, ^•^ p < 0.1
